# Implicit Attitudes to Female Body Shape in Spanish Women With High and Low Body Dissatisfaction

**DOI:** 10.3389/fpsyg.2019.02102

**Published:** 2019-09-18

**Authors:** Mónica Hernández-López, Alba Antequera-Rubio, Miguel Rodríguez-Valverde

**Affiliations:** Department of Psychology, University of Jaén, Jaén, Spain

**Keywords:** implicit attitudes, weight bias, body image, body dissatisfaction, implicit relational assessment procedure, IRAP

## Abstract

Research on implicit attitudes to body image has grown substantially in recent years. The extant evidence reveals an implicit weight bias in the general population that has generally been interpreted in terms of anti-fat attitudes. However, research with the Implicit Relational Assessment Procedure (IRAP) shows that this bias appears to be driven by pro-slim rather than anti-fat implicit attitudes. Besides, the only IRAP study of this sort conducted in Spain found no evidence of such implicit weight bias (with similarly positive attitudes to thinness and fatness). Given the existing differences in body dissatisfaction (BD) among diverse cultural contexts, we predicted that discrepancies in findings about implicit weight bias might be related to differences in BD amongst the samples in the different studies. This study explores whether women with extreme scores in BD (High vs. Low) show different patterns of attitudes to female body shape. Spanish female college students with extreme scores in the Body Shape Questionnaire (BSQ: high ≥ 104, percentile 80; low ≤ 52, percentile 20) completed an IRAP with pictures of overweight and underweight women as target stimuli and the words pleasant and unpleasant as labels. Participants also completed explicit ratings to the same stimuli and clinically relevant measures of body image related distress. Results showed an implicit weight bias only for women high in BD. While both groups showed equally positive implicit attitudes to thinness, only women with low BD showed implicit positive attitudes to fatness (and hence no bias). In turn, both groups presented a clear pro-thin/anti-fat explicit bias with positive ratings for underweight pictures and negative ratings for overweight pictures. The latter were stronger for the high BD group. Therefore, between-group differences were mainly driven by differences in attitudes to fatness (both implicit and explicit). Both implicit and explicit attitudes to fatness independently predicted eating disorders symptoms and other clinically relevant measures. These results are discussed in terms of their clinical implications.

## Introduction

Body dissatisfaction (BD) can be defined as a subjective disapproval of one’s own body shape and the belief that it is unattractive to others (Ferguson, 2013). BD and more specifically the desire for thinness, is widespread especially in Western societies (Frederick et al., 2006, 2007; Swami et al., 2010). Research carried out in the last 20 years has shown that more than half of women are dissatisfied with their bodies, that a large majority of them would desire to lose weight, and some of them would be open to giving up years of life in exchange for the desired body shape (Garner, 1997; Monteath and McCabe, 1997; Bearman et al., 2006). The high prevalence of BD, especially in young women, has been attributed to the globalization of Western cultural values (Warren et al., 2005) and more specifically to the continuous exposure to thin-ideal images through the mass media (Swami et al., 2010). BD is considered a key construct in body image research because of its association with unhealthy eating habits and its role as a risk factor for the emergence and maintenance of eating disorders (Stice and Shaw, 2002). It also shows clear associations with other clinically relevant constructs in body image research (e.g., body-image related psychological inflexibility: Sandoz et al., 2013). While it is assumed that BD is widespread and appears to be normative worldwide nowadays, the picture is complex. Most research has been conducted in Western English-speaking countries, where exposure to the thin ideal is extensive. However, recent cross-cultural research has also shown differences in levels of BD amongst women from different countries, ethnicities and cultural backgrounds (Swami et al., 2010).

Despite its indubitable relevance, BD has received limited attention in research on attitudes to body size, a field that has grown substantially in the last years (e.g., Rozin and Fallon, 1988; Schwartz et al., 2003; Brownell et al., 2005). Substantial accumulated evidence supports the view that there is a pro-thin/anti-fat attitudinal bias in the general population. Individuals with overweight and obesity are generally considered to be less successful, attractive and competent than thin people (Crandall, 1994; Teachman et al., 2003; Schwartz et al., 2006). A substantial part of these research findings have been collected through the use of questionnaires and other self-report measures (e.g., Crandall, 1994). In spite of their utility and convenience, the extent to which these instruments are adequate for the assessment of attitudes toward socially controversial issues has been put into question (e.g., Dovidio et al., 1997). They are potentially susceptible to self-presentation management and demand characteristics (Holtgraves, 2004). Furthermore, it is not clear to what extent participants can accurately introspect on their social biases (Nisbett and Wilson, 1977; Wilson, 2009).

Implicit measures can address some of the limitations of self-report. Typically, implicit measures require participants to rapidly and accurately respond to stimuli presented on a computer under time-pressure conditions. Participant’s immediate, automatic attitudes are inferred from their task performance. The most commonly employed implicit measure in body image research is the Weight Implicit Association Test (Weight IAT) (see Teachman and Brownell, 2001; Teachman et al., 2003; Ahern and Hetherington, 2006; Gapinski et al., 2006; Schwartz et al., 2006; Brochu and Morrison, 2007), a version of the Implicit Association Test (IAT: Greenwald et al., 1998). Most studies that have employed the Weight IAT generally find a clear pro-thin/anti-fat implicit attitudinal bias that seems to be stronger than that observed with explicit measures (Teachman et al., 2003; Schwartz et al., 2006). In the Weight IAT, two body size categories (e.g., Thin vs. Fat) and two classes of attribute stimuli (e.g., Good vs. Bad) are presented together in each trial. Attitudinal biases are inferred from differences in response latencies to differently conformed trials (Thin-Good/Fat-Bad and Thin-Bad/Fat-Good) but these latencies always reflect sorting a stimulus to one body size-attribute pair relative to another body size-attribute pair, rather than independently of each other (De Houwer, 2002; Hughes et al., 2011). Given this task structure, the IAT only yields a relative measure of preference for one category over the other (De Houwer, 2002), and does not allow (with ordinary scoring and analysis methods) for knowing the direction of the observed implicit bias.

A recent study (Anselmi et al., 2013) addressed this limitation of the Weight IAT by using a Rasch analysis that disentangled the separate contribution of positive and negative associations (amongst images of faces of fat and thin people and evaluative terms) to the overall measure. Results showed a clear typical Weight IAT effect (people were faster when they had to associate Thin-Good/Fat-Bad than when they had to associate Fat-Good/Thin-Bad), but this effect was mostly explained by Thin-Positive associations, meaning that implicit weight bias is driven by a positive evaluation of thin people and that it does not necessarily involve a derogation of fat people. These findings are consistent with the outcomes of other studies that have employed a newer procedure for the measurement of implicit attitudes, the Implicit Relational Assessment Procedure (IRAP: Barnes-Holmes et al., 2006).

The IRAP is a computerized reaction time-based procedure grounded on a functional-contextual model of human language and cognition (Relational Frame Theory: see Hayes et al., 2001; see also Hughes and Barnes-Holmes, 2013). From this perspective, it is assumed that participants have a long pre-experimental learning history of relating the concepts that are presented in the task. The IRAP assesses how participants respond to different types of relations amongst these concepts, with the assumption that participants will be faster in responding to the types of relations that are consistent with their learning history. Procedurally, there is a crucial difference between the IAT and the IRAP. Unlike the IAT, in each trial the IRAP only presents one category (e.g., either Thin or Fat) and one attribute (e.g., either Good or Bad) and two response options (e.g., True and False), which renders four different trial types (Thin-Good; Fat-Bad; Thin-Bad; Fat-Good). Instead of alternating differently conformed blocks of trials (e.g., Thin-Good/Fat-Bad vs. Thin-Bad/Fat-Good), the IRAP presents alternating blocks of the same four trial types, only differing in the relational response required in each block. For instance, in pro-thin/anti-fat blocks, participants are required to respond True to Thin-Good and Fat-Bad trial types, and to respond False to Thin-Bad and Fat-Good trial types. In anti-thin/pro-fat blocks, the required responses are the opposite for each trial type. This allows the IRAP to offer a non-relative measure of implicit attitudes, with the possibility of calculating specific scores for each trial type or for each category (Thin vs. Fat), in addition to a single overall, relative preference score.

The conceptual rationale underlying the IRAP argues that implicit and explicit measures lead to different patterns of relational responding. According to the Relational Elaboration and Coherence model (Barnes-Holmes et al., 2010; Hughes et al., 2011), the different behavioral effects captured by implicit (e.g., reaction-time based tasks) and explicit (e.g., questionnaires) procedures reflect the operation of the same behavioral process (i.e., arbitrarily applicable relational responding). However, depending on the properties of each assessment situation (e.g., time-pressured or not), two broad patterns of relational responding can be obtained. One, brief and immediate, reflects a strongly reinforced verbal response involving the stimuli presented in the task. The other, extended and elaborated, entails additional relational responding not only to the stimuli themselves, but also to the initial response to those stimuli. Without time constraints, these additional relational responses will form a relational network that has to be coherent and consistent with other existing relational networks. For instance, a participant’s immediate reaction to a picture of an underweight woman in the IRAP might be an automatic evaluation of it as attractive (given extensive media exposure to thin models as beauty ideals). However, when asked to rate the same picture without time constraints to respond, other more elaborate verbal relations might emerge (e.g., “extreme thinness is dangerous for health,” “I am not a person who judges others based on appearance”).

So far, no study conducted with the IRAP has reported clear evidence of anti-fat implicit attitudes, either when assessing other-related (Roddy et al., 2010, 2011; Nolan et al., 2013; Maroto-Expósito et al., 2015) or self-related attitudes (Parling et al., 2012; Ritzert et al., 2016). Different studies conducted with Irish female and male participants have found clear positive attitudes to average-weight pictures and neutral attitudes to overweight pictures (e.g., Roddy et al., 2010, 2011). However, a very similar study conducted with a female-only sample of Spanish college students (Maroto-Expósito et al., 2015) revealed a different pattern of results. In this case, no implicit bias (i.e., relative preference) was found for pictures of thinness compared to pictures of fatness. Results indicated an implicit positive attitude of similar magnitude to both types of pictures (and hence no implicit weight bias at all). In addition, participants showed a clear and strong pro/thin and anti/fat explicit bias measured with visual analog rating scales for the pleasantness of each picture presented in the IRAP (i.e., clear positive ratings of thin pictures and clear negative ratings of overweight pictures). The absence of an implicit weight bias and the finding of a strong pro-thin/anti-fat explicit bias in that study is clearly at odds with the general literature on implicit weight-bias. These discrepancies between the results of studies conducted in different countries could be interpreted in light of cultural differences between samples. The few studies on anti-fat attitudes conducted with Spanish samples report smaller bias than similar studies from the United States or from Northern Europe (see Solbes and Enesco, 2010). There is also evidence that, while Spanish females rate their actual and ideal body size similarly to European-American females, the latter are more dissatisfied with their bodies than the former (Gleaves et al., 2000). Specifically, Spanish females score substantially lower on body dissatisfaction as measured with the Body Shape Questionnaire (BSQ) than European-American females (see Warren et al., 2008). In our view, these findings can be taken as indicative that a smaller concern for others’ body size (as reflected by weaker weight bias) is associated with less dissatisfaction with one’s own body and, more generally, with less body-image related distress. The present study was devised to explore the idea that different levels of BD are associated to different patterns of implicit attitudes to others’ body size as measured with the IRAP.

The main aim of the present study was to explore whether Spanish women high or low in BD differed in their implicit and explicit attitudes to female pictures of underweight and overweight. A secondary aim was to explore whether implicit and explicit attitudes to others’ body size were predictive of body-image related distress. We expected participants high in BD to show an implicit relative preference for pictures of thinness over those of fatness. In contrast, we expected to find no such relative preference for women low in BD. We also expected that this between-group difference would be due to attitudes to pictures of fatness, more than to attitudes to thinness (given that all prior studies with the IRAP have consistently found pro-thin implicit attitudes, and the different findings amongst published studies specifically regard attitudes to fatness). Regarding explicit ratings, we expected a positive evaluation of thinness and a negative evaluation of fatness in both groups (like in the only previous study with Spanish population, Maroto-Expósito et al., 2015), with a stronger negative evaluation of fatness for women high in BD. According to the extant IRAP literature on weight bias, we hypothesized an absence of correlation between IRAP scores and explicit ratings of the same target pictures.

## Materials and Methods

### Participants

Four hundred and twenty-eight female students at a mid-sized public university in Southeastern Spain completed the Body Shape Questionnaire (BSQ; Cooper et al., 1987) as part of a screening that involved different questionnaires. Participants with high levels of BD (BSQ score ≥ 104, 80th percentile of the sample’s BSQ score distribution) and low levels of BD (BSQ score ≤ 52, 20th percentile) were invited to participate in the second part of the study, consisting of an assessment of implicit and explicit body size attitudes. Ninety-three of them agreed to participate in the experimental session and were assigned to either of the two groups (High vs. Low dissatisfaction), but 41 did not fulfill the IRAP criteria (see procedure) and were excluded. The final sample consisted of 52 participants [26 with high BD (mean BSQ score = 126.35, *SD* = 17.40) and 26 with low BD (mean BSQ score = 44.54, *SD* = 5.92)] with ages between 18 and 39 years old (*M* = 20.60; *SD* = 3.29), average body mass index (BMI) in the range of normal weight (*M* = 21.59; *SD* = 3.69), and no self-reported cases of eating disorders (ED) or other severe psychopathologies. Participants had no prior experience with implicit measures. They received course-credit for participation in the study. The sample size in this study fulfills recommendations for the benchmark statistical power of 0.80 for medium-to-large effect sizes, according to a recent meta-analysis of the IRAP in the clinical domain (see Vahey et al., 2015).

### Measures

#### Demographics and Anthropometric Measures

A paper-and-pencil form with open-ended questions about age, education, current or past diagnoses of eating disorders or other severe psychological disorders, weight and height. Body-mass index (weight/height^2^) (kg/m^2^) was calculated for each participant based on self-reported weight and height.

#### Body Shape Questionnaire (BSQ; Cooper et al., 1987)

The BSQ comprises 34-items that assess dissatisfaction with one’s own body-shape. The items are answered on a 6-point Likert scale (1 never, 6 always). In this study we used the Spanish version of the BSQ (Raich et al., 1996), which has shown good psychometric properties, with high internal consistency (in the current study, Cronbach’s α = 0.97), and good concurrent validity with other eating disorder measures (Warren et al., 2008). The clinical cutoff score for Spanish population is 105 (see Raich et al., 1996).

#### The Eating Attitudes Test (EAT-40; Garner and Garfinkel, 1979; Garner et al., 1982)

The EAT-40 is a 40-item questionnaire that assesses symptoms of eating disorders (anorexia and bulimia nervosa). Each statement is answered on a 6-point Likert scale, with the three highest options (always, usually, often) respectively scoring 3, 2, or 1 points, and the remaining three options scoring 0 points. The Spanish version (Castro et al., 1991) used in this study has shown good psychometric properties (Cronbach’s α = 0.89 in the current study).

#### Body Image-Acceptance and Action Questionnaire (BI-AAQ; Sandoz et al., 2013)

The BI-AAQ is a 12-item self-report scale that assesses psychological inflexibility regarding one’s body image, that is, unwillingness to experience unwanted thoughts and emotions about one’s body shape and/or weight. Items are rated on a 7-point Likert scale (1: never true, 7: always true). We translated the original questionnaire into Spanish and the measure revealed good psychometric properties (Cronbach’s α = 0.95).

#### Implicit Relational Assessment Procedure (IRAP)

The IRAP is a reaction-time based computerized tool for the direct assessment of brief, immediate relational responding (we used the IRAP version 2012 programed in Microsoft Visual Basic 6.0). The words “Pleasant” and “Unpleasant” were used as samples or label stimuli. The target stimuli were twelve photographs, each of them depicting one different young woman: six portrayed underweight women (BMI < 18.5) and the other six portrayed overweight women (BMI > 25). These categories are in accordance with the World Health Organization BMI classification (World Health Organization [WHO], 1995). All models were undergraduate volunteers (from a different university to the one where the study was conducted). Each photo was a full-body picture of a woman standing up in the middle of the image, arms close to her body and her face pixelated (in order to keep models anonymous and to prevent differences in attractiveness between pictures). In all of them, models were similarly dressed with jeans and a top that allowed the viewer to easily perceive their silhouette. Each woman appeared against the same white neutral background so as to minimize any distracting elements. The size of each picture was 432 × 576 pixels (72 ppi) with RGB color. These photos were used in previous research with good results as to their discriminability based on body shape (see Maroto-Expósito et al., 2015). Additionally, in each IRAP trial, participants were presented with two relational response options, “True” and “False” (for more details, see Procedure).

#### VAS

Visual Analogue Scales (VAS) were employed for the assessment of explicit attitudes to the same photos used in the IRAP. Each scale comprised a 100 mm line with the words “Pleasant” and “Unpleasant” below the right and left ends of the line, respectively. Ratings (0–100 mm) were transformed in order to make the explicit scores comparable to the scores produced by the IRAP (with positive scores indicating pro-slim/anti-fat attitudes and negative scores indicating pro-fat/anti-slim attitudes). For photos of underweight, ratings ranged from −50 (unpleasant) to +50 (pleasant), while for photos of overweight, they ranged from +50 (unpleasant) to −50 (pleasant).

### Procedure

All procedures were approved by the University Ethics Review Board. Participant recruitment was conducted through in-class announcements. Participants were informed that the study included two different phases. In the first one, they answered a series of questionnaires about body image. Participants selected upon their BSQ score (≤52 or ≥104) were contacted by phone and invited to take part in the second phase. This phase consisted of the completion of the IRAP followed by completion of the VAS.

#### Phase 1

Participants signed a statement of informed consent upon explanation of the study features. They underwent the procedure in group, in a class at the University. They were requested to respond sincerely and reminded that all the information provided would be confidential and anonymous. Measures were presented in paper-pencil format in the following order: BSQ, EAT, BI-AAQ and the demographic and anthropometric measures. The procedure took around 15 min.

#### Phase 2

Participants underwent the procedures of this phase individually in a sound-attenuated experimental cubicle equipped with Windows-based computer. They completed the IRAP task first and the VAS afterward.

The IRAP program began with the on-screen presentation of task instructions. Comprehension was assessed by requesting participants to briefly explain the task. After any doubts were clarified the task began. It was a typical IRAP preparation with trial-blocks presented in pairs, comprising a practice (minimum one pair of blocks) and a test phase (fixed to three pairs of blocks). One block of each pair was pro-slim/anti-fat and the other was pro-fat/anti-slim. Standard specific criteria (80% correct responding and a median latency under 2000 ms) had to be achieved for each practice block (of the same pair) in order to advance to the test blocks. Participants failing to meet the criteria on the first pair of practice blocks were re-exposed to practice blocks up to a maximum of six pairs. Upon failing to reach criteria after this exposure they were discarded. The same accuracy and latency criteria applied for test blocks (although in this case participants completed the fixed sequence of three block pairs regardless of their performance). Data from participants failing to maintain criteria in any of the six test blocks were also discarded. Half of the participants in each group began the task with a pro-slim/anti-fat block, followed by a pro-fat/anti-slim block (and all subsequent blocks, either practice or test, alternated according to this sequence). The other half underwent the task with the opposite sequence: pro-fat/anti-slim first, then pro-slim/anti-fat.

**FIGURE 1:**
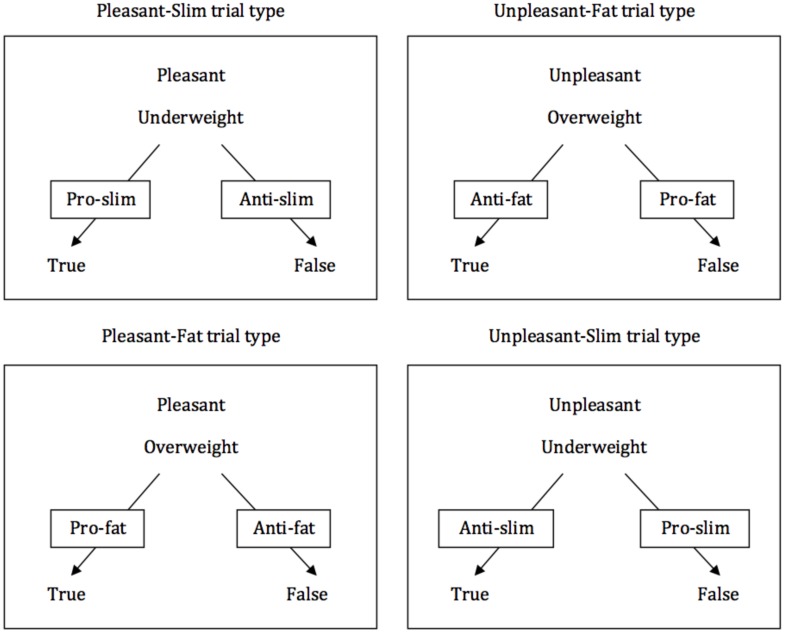
Representations of the four IRAP trial-types. The attribute label stimulus (“Pleasant” or “Unpleasant”) appeared at the top of the screen while the target stimulus (a photo of either an underweight or an overweight young woman) appeared in the middle of the screen. The response options “True” and “False” appeared simultaneously on each trial at the bottom of the screen. The arrows and the labels superimposed on them indicate, for each trial-type, the correct response in either pro-slim/anti-fat blocks or in pro-fat/anti-slim blocks (the boxes and arrows did not appear on screen on actual trials during the task, and they have been included here for illustration purposes only).

Each block comprised 24 trials, resulting from the combination of the two labels (“Pleasant” and “Unpleasant”) and the twelve target photos. Trials were presented in quasi-random order, with no more than two consecutive presentations of the same type of trial (e.g., Pleasant-Underweight). In each trial, the label appeared at the top center of the screen and the target photo appeared under the label. The response options “True” and “False” appeared one on the left and the other on the right side of the screen bottom. Participants had to respond by pressing the keyboard keys “d” (for the option on the left) or “k” (for the option on the right). Across trials, response options were randomly allocated (left or right) with a maximum two consecutive trials on the same positions. If participants took longer than 2000 ms to respond in any given trial, feedback (the words “very slow”) appeared immediately and remained on screen until the participant produced a response. Incorrect responses produced a red “X” that remained in the middle of the screen until production of the correct response. Correct responses started a 400 ms blank-screen inter-trial interval followed by a new trial. The task comprised four different trial-types: the label “Pleasant” and a photograph of an underweight woman; the label “Unpleasant” and a photograph of an underweight woman; the label “Pleasant” and a photograph of an overweight woman; and the label “Unpleasant” and a photograph of an overweight woman (see Figure 1). In pro-slim/anti-fat blocks, participants had to press the key for “True” in trials presenting the combinations Pleasant-Underweight and Unpleasant-Overweight, and “False” in trials presenting the combinations Unpleasant-Underweight and Pleasant-Overweight. In pro-fat/anti-slim blocks, they were required to respond in accordance with the opposite pattern. Each block finished with the onscreen presentation of feedback (median latency and percentage of correct responses in the block). Participants then proceeded to the next block with the instruction to respond in the opposite manner to their responding in the previous block.

The only differences between practice and test blocks were: (a) participants received a more complete feedback reminding them of the required criteria after each pair of practice blocks, and (b) while practice blocks started with the instruction “This is practice. It is normal to make mistakes. Please try to respond quickly and correctly” test blocks began with the instruction “This is a test, try to respond quickly and correctly.”

Upon termination of the IRAP, participants completed the VAS. The same 12 target pictures used with the IRAP were presented on the screen, one at a time, according to a quasi-random sequence with the limitation that a picture from each body size category (underweight vs. overweight) was not presented more than twice consecutively. Participants had no time limits to rate each picture on the VAS. Once they rated a picture, they advanced to the next by pressing a key in the keyboard. The complete procedure took around 30 min.

### Data Preparation

Participants who failed to achieve the accuracy and latency criteria during the practice phase (19 out of 93) or to maintain such criteria in any of the IRAP test blocks (22 of the 74 that had passed the practice phase) were excluded. The remaining participants (52) had their IRAP performance quantified and analyzed. Response latency (time elapsed, in ms, between the onset of visual stimulus presentation on screen and the emission of a correct response by the participant) is the primary datum of the IRAP. Following standard practice, the *D*-IRAP score, an adaptation of the *D*_1_-algorithm (Greenwald et al., 2003), a variant of Cohen’s *d* (see Barnes-Holmes et al., 2009, for a detailed description), was used to quantify the magnitude of differences in response latencies between both IRAP conditions (pro-slim/anti-fat vs. anti-slim/pro-fat). The steps for this calculation were as follows: (1) only latency data from the six test blocks were used; (2) latencies over 10000 ms were removed from the dataset (0% latencies removed); (3) all data from any single participant were removed if they had more than 10% of test-block trials with latencies less than 300 ms (0% participants lost to this step); (4) three standard deviations were computed for each of the four trial types (making a total of 12 SDs): four for all latencies in test blocks 1 and 2, four for test blocks 3 and 4, and four for test blocks 5 and 6; (5) for each of the six test blocks, four mean latencies were calculated, one per trial type; (6) for each pair of test blocks and trial type, the mean latency of the pro-slim/anti-fat block was subtracted from the mean latency of the corresponding pro-fat/anti-slim block, yielding 12 difference scores (one per trial type and pair of blocks); (7) each difference score was divided by its corresponding standard deviation from step 4, yielding 12 *D*-IRAP scores; (8) the three scores for each trial type (one per test block pair) were averaged to produce a single *D*-IRAP score per trial type; (9) the *D*-IRAP scores for the two trial types presenting images of slimness (*Pleasant-Underweight* and *Unpleasant-Underweight*) were averaged, and the same was done for the scores of trial types *Pleasant-Overweight* and *Unpleasant-Overweight*, which produced a single *D*-IRAP score to each body size category (*D*_*slim*_ and *D*_*fat*_); (10) an overall relative *D*-IRAP score (*D*_*general*_) was calculated by averaging *D*_*slim*_ and *D*_*fat*_. Positive *D*_*general*_ scores were indicative of a general pro-slim/anti-fat bias, and negative ones were indicative of general pro-fat/anti-slim bias. Positive *D*_*slim*_ scores were indicative of a specific pro-slim attitude, and negative scores were indicative of a specific anti-slim attitude. Positive *D*_*fat*_ scores were indicative of a specific anti-fat attitude, and negative scores were indicative of a specific pro-fat attitude.

## Results

**FIGURE 2:**
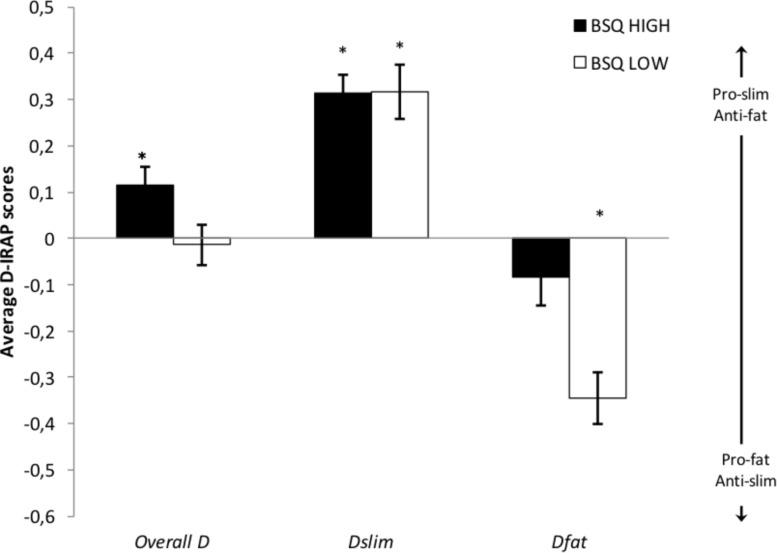
Mean (plus/minus s.e.m.) overall (all trial types) and specific *D*-IRAP scores (*D*_*slim*_ for trials presenting pictures of underweight; *D*_*fat*_ for trials presenting pictures of overweight). Positive scores are pro-slim/anti-fat, and negative scores are pro-fat/anti-slim. Asterisks indicate that the score is significantly different from zero (*p* < 0.05).

For each group, three one-sample *t*-tests were conducted to determine if each of these measures was significantly different from zero. Figure 2 presents the averages for each *D*-IRAP score per group. The *D*_*general*_ score (averaging all four trial-types) for the high dissatisfaction group was 0.114 (*SD* = 0.211), and it was significantly different from zero (*t*_(25)_ = 2.761, *p* = 0.011, *d* = 0.540), which would be indicative of a significant general weight bias. The specific *D*-IRAP scores for this group indicate that this general bias is exclusively attributable to a pro-thin attitude. The *D*_*slim*_ score (*M* = 0.314; *SD* = 0.203) was significantly different from zero (*t*_(25)_ = 7.866, *p* < 0.001, *d* = 1.547), while the *D*_*fat*_ score (*M* = −0.085; *SD* = 0.308) was not (*t*_(25)_ = −1.407, *p* = 0.172, *d* = 0.276). Accordingly, participants in this group showed positive implicit attitudes to pictures of underweight women, and a neutral attitude (neither positive nor negative) to pictures of overweight women (i.e., a pro-slim implicit bias). For the low dissatisfaction group, the *D*_*general*_ score (*M* = −0.014; *SD* = 0.223) was not significantly different from zero (*t*_(25)_ = −0.321, *p* = 0.751, *d* = 0.063). Both specific *D*_*slim*_ (*M* = 0.316; *SD* = 0.299) and *D*_*fat*_ scores (*M* = −0.344; *SD* = 0.284) were significantly different from zero (*D*_*slim*_: *t*_(25)_ = 5.375, *p* < 0.001, *d* = 1.057; *D*_*fat*_: *t*_(25)_ = −6.185, *p* < 0.001, *d* = 1.211), which reveals positive implicit attitudes (of similar magnitude) to both underweight and overweight pictures, and thus an absence of implicit weight bias.

To determine whether *D*-IRAP scores were dependent on BD, a 2 (BD: High vs. Low) × 2 (*D*-IRAP: *D*_*slim*_ vs. *D*_*fat*_) repeated measures (Greenhouse-Geisser-corrected) ANOVA was conducted. In addition to a significant within-subject effect for the *D*-IRAP score type (*F*_(1, 50)_ = 123.251, *p* < 0.001, η^2^_p_ = 0.711), both a significant interaction, *F*_(1, 50)_ = 7.527, *p* = 0.008, η^2^_p_ = 0.131, and a significant between-group main effect were found, *F*_(1, 50)_ = 4.542, *p* = 0.038, η^2^_p_ = 0.083. Additional planned comparisons (independent-samples *t* tests) revealed a significant difference between groups for the *D*_*fat*_ (*t*_(50)_ = 3.154, *p* = 0.003, *d* = 0.892) but not for the *D*_*slim*_ score (*t*_(50)_ = −0.036, *p* = 0.972, *d* = 0.001).

**FIGURE 3:**
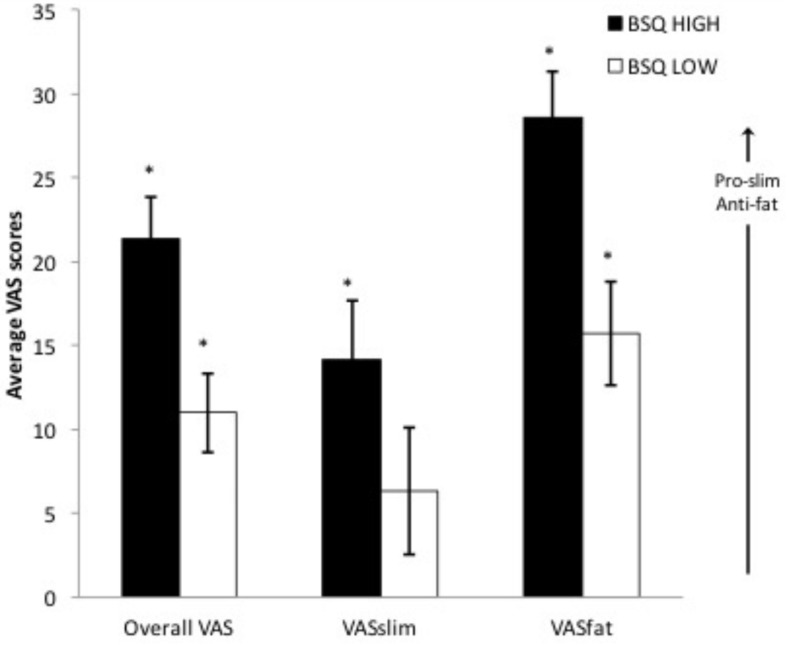
Mean (plus/minus s.e.m.) explicit overall (all trial types) and specific VAS ratings (VAS_slim_ for pictures of underweight; VAS_fat_ for pictures of overweight). Positive scores are pro-slim/anti-fat. Asterisks indicate that the score is significantly different from zero (*p* < 0.05).

Figure 3 presents average explicit VAS-based ratings for both groups and types of pictures, as well as an average overall VAS score for each group. For both groups, all ratings were positive and thus indicative of explicit pro-slim/anti-fat bias. With the exception of VAS_slim_ scores for the low BD group (*t* = 1.661, *p* = 0.109), all other VAS scores were significantly different from zero (smallest *t* = 3.989, *p* = 0.001; smallest *d* = 0.782). Participants in both groups showed explicit negative attitudes to pictures of overweight women. In addition, participants in the high BD group showed explicit positive attitudes to pictures of underweight women. For the high BD group both ratings (VAS_slim_: *M* = 14.157; *SD* = 18.096; VAS_fat_: *M* = 28.630; *SD* = 13.571) were larger than for the low BD group (VAS_slim_: *M* = 6.316; *SD* = 19.392; VAS_fat_: *M* = 15.715; *SD* = 15.622). A 2 (BD: High vs. Low) × 2 (VAS rating: VAS_slim_ vs. VAS_fat_) repeated measures ANOVA showed significant within-subject (*F*_(1, 50)_ = 14.299, *p* < 0.001, η^2^_p_ = 0.222) and between-group (*F*_(1, 50)_ = 9.165, *p* = 0.004, η^2^_p_ = 0.155) effects, but no significant interaction (*F*_(1, 50)_ = 0.643, *p* = 0.426, η^2^_p_ = 0.013). Given our interest in exploring potential between-group differences in specific attitudes toward thinness and fatness, we conducted further independent samples *t* tests as planned comparisons that revealed a significant between-group difference for VAS_fat_ (*t*_(50)_ = 3.182, *p* = 0.003, *d* = 0.883) but not for VAS_slim_ (*t*_(50)_ = 1.507, *p* = 0.138, *d* = 0.418) ratings.

Correlations between each specific *D*-IRAP score, VAS score and each self-report measure (BSQ, EAT, and BI-AAQ) and BMI were calculated (see Table 1). Given that the sample in this study consisted of participants showing either a very high or a very low level of BD, most variables were not normally distributed. Accordingly, following Heider et al. (2015, p. 5) Spearman’s rank-order correlation coefficients were calculated instead of Pearson product-to moment correlation coefficients. There was a positive correlation between the implicit scores obtained only from trials presenting photos of underweight women (*D*_*slim*_), and the explicit ratings of pleasantness for this type of pictures (VAS_slim_ scores). No other significant correlations were present for either of these variables. In turn, implicit scores drawn only from trials presenting photos of overweight women (*D*_*fat*_ scores) did not correlate with any of the explicit VAS ratings. However, *D*_*fat*_ scores correlated positively with BD, eating disorder symptoms and body-image psychological inflexibility. That is, women with higher levels of dissatisfaction with their own body, with stronger eating disorder symptoms, and less accepting of their body image showed stronger anti-fat/weaker pro-fat implicit attitudes to images of overweight women. Similar positive correlations were observed for explicit ratings of pictures of overweight women (VAS_fat_ scores). Interestingly, both implicit and explicit attitudes toward pictures of overweight correlated positively with measures of body image related distress. However, these two variables show a lack of correlation between them (ρ = −0.004).

**TABLE 1:** Spearman’s rank-order correlation coefficients (ρ) between *D*-IRAP scores and explicit measures (*N* = 52).

	**BMI**	***D*_*slim*_**	***D*_*fat*_**	**VAS_slim_**	**VAS_fat_**	**BSQ**	**EAT-40**	**BI-AAQ**
BMI	–							
*D*_*slim*_	0.191	–						
*D*_*fat*_	0.249	0.236	–					
VAS_slim_	−0.160	0.390^∗∗^	0.089	–				
VAS_fat_	−0.050	−0.062	−0.004	0.200	–			
BSQ	0.316^∗^	0.054	0.419^∗∗^	0.231	0.358^∗∗^	–		
EAT-40	0.239	0.000	0.331^∗^	0.220	0.412^∗∗^	0.849^∗∗^	–	
BI-AAQ	0.165	−0.013	0.342^∗^	0.205	0.425^∗∗^	0.843^∗∗^	0.863^∗∗^	–

*BMI, body mass index; VAS, Visual Analog Scale; BSQ, Body Shape Questionnaire; EAT, Eating Attitudes Test; BI-AAQ, Body Image Acceptance and Action Questionnaire. ^∗^*p* < 0.05; ^∗∗^*p* < 0.01.*

In order to explore whether implicit and explicit attitudes toward pictures of overweight women could be used to make clinically relevant predictions regarding body image, we conducted hierarchical regression analyses with eating disorder symptoms (EAT-40 scores), BD (BSQ scores) and body image psychological inflexibility (BI-AAQ scores) as the criterion variables. For each model, VAS_fat_ scores were introduced in the first step and *D*_*fat*_ scores were introduced second. For all dependent variables VAS_fat_ scores proved to be a statistically significant predictor, and *D*_*fat*_ scores produced a significant increment in predictive validity (see Table 2). Overall, regression analyses showed that both implicit and explicit attitudes to pictures of overweight women independently contributed to predicting clinically relevant measures of body-image related distress.

**TABLE 2:** Hierarchical regression predicting eating disorder symptoms (EAT-40), body-image related psychological inflexibility (BI-AAQ), and body dissatisfaction (BSQ) from explicit (VAS_fat_) and implicit (*D*_*fat*_) attitudes to fatness.

	***B***	**SE**	**β**	***F***	***R*^2^**	**Δ*R*^2^**
**Dependent variable: EAT-40**						
Step 1				8.955^∗∗^	0.152	–
VAS_fat_	0.474	0.159	0.390			
Step 2				7.570^∗∗^	0.236	0.084
VAS_fat_	0.462	0.152	0.380			
*D*_*fat*_	17.483	7.525	0.290			
**Dependent variable: BI-AAQ**						
Step 1				11.473^∗∗^	0.187	–
VAS_fat_	0.556	0.164	0.432			
Step 2				10.079^∗∗∗^	0.291	0.105
VAS_fat_	0.541	0.155	0.421			
*D*_*fat*_	20.618	7.657	0.324			
**Dependent variable: BSQ**						
Step 1				10.667^∗∗^	0.176	–
VAS_fat_	1.142	0.350	0.419			
Step 2				13.409^∗∗∗^	0.354	0.178
VAS_fat_	1.103	0.313	0.405			
*D*_*fat*_	56.858	15.482	0.422			

*^∗^*p* < 0.05; ^∗∗^*p* < 0.01; ^∗∗∗^*p* < 0.001.*

## Discussion

Consistent with our initial hypothesis, the results in this study show a clear differential pattern of implicit body-shape attitudes in Spanish female college students reporting high or low levels of BD. Participants with low BD showed no relative preference for one type of pictures over the other, with implicit positive attitudes of equal magnitude to photographs of both women with underweight or overweight. These results are similar to the ones found by Maroto-Expósito et al. (2015) for a general sample of Spanish female college students with average BSQ scores. In contrast, participants with high BD showed a relative preference for images of thinness compared to images of fatness, which is similar to that observed in previous studies with the IRAP conducted with Irish participants (Roddy et al., 2010, 2011). This implicit relative preference is attributable to pro-slim implicit attitudes, and not to anti-fat ones. These findings reveal that self-reported levels of BD are clearly associated with implicit body-image attitudes toward other females’ body shape as measured by the IRAP. Given that previous studies (Roddy et al., 2010, 2011; Nolan et al., 2013) on the topic did not collect information on participants’ levels of BD, it is difficult to elucidate whether inconsistent findings across studies (cf. Roddy et al., 2010; Maroto-Expósito et al., 2015) are attributable to differences in BD across samples from different cultural contexts. It cannot be excluded either that differences in findings among these studies might be related with differences in sample composition (i.e., males and females compared to a female-only sample) and the target stimuli employed (i.e., images of both males and females compared to female-only images) (for a detailed discussion see Maroto-Expósito et al., 2015). In any case, the findings in the present study suggest that future studies on implicit attitudes to body size should take participants’ levels of BD into account.

The fine-grained analysis that the IRAP allows (with specific measures for each body size category) provides more information than it would be possible to obtain with other implicit procedures like the IAT. The specific *D*_*fat*_ and *D*_*slim*_ scores show that between-group differences were entirely based on different implicit attitudes to fatness. While both groups showed equally positive attitudes to pictures of underweight women, they differed in their response to pictures of overweight women, with women low in dissatisfaction presenting a clear positive attitude to them and women high in dissatisfaction showing a neutral attitude. Previous research with the IRAP (including this very study) reveals that implicit weight-bias (when it appears at all) is driven by pro-slim attitudes. However, this study shows that the key difference in implicit weight bias between two very different groups in terms of a clinically relevant body-image feature stems from differences in their implicit evaluation of fatness, not thinness. This is interesting, because research on the negative psychological impact of exposure to thin ideal images shows that BD appears to mediate such impact, constituting a form of vulnerability. Women with pre-existing higher levels of BD show stronger increases in BD after exposure to pictures of thin and underweight females than women with previous lower levels of BD (Groesz et al., 2002; Ferguson, 2013). In this case, regarding implicit attitudes, we have not found that higher BD entails a more positive automatic evaluation of thinness. It could be hypothesized that widespread and continued exposure to thin ideal images leads to positive implicit evaluation of thinness by women in general, regardless of how happy they are with their own body. In turn, it seems that only the women who show a similar appreciation for a larger body shape would be content with their own body. In summary, having a positive outlook that is constrained to images of underweight or extreme thinness (and not open to other body shapes) might be a marker for body dissatisfaction and in turn for risk of eating disorders. In any case, further research is needed in order to better understand the association between implicit weight bias and these clinically relevant constructs.

In principle, results obtained with the VAS present a different picture, with positive attitudes to thinness and clearly negative attitudes to fatness in both groups. That is, the observed explicit weight bias has both a pro-slim and an anti-fat component. This general tendency is stronger for women highly dissatisfied with their bodies. This pattern of results indicating stronger explicit than implicit weight bias is similar to that found in Maroto-Expósito et al. (2015), but uncommon in the literature on body-size attitudes, where stronger bias is usually found for implicit than for explicit attitudes (Teachman et al., 2003; Roddy et al., 2010, 2011; Nolan et al., 2013). In our view, this discrepancy in the findings may be related to the fact that only the Maroto-Expósito et al. study and this one have employed as an explicit measure a VAS for the same target images presented in the IRAP. Instead, other studies asked participants for generic evaluative responses to the relatively general categories of “thin people” and “overweight people.” The VAS might have been less susceptible to social desirability effects than other frequently employed explicit measures. Also, the fact that the study was conducted in a non-clinical context and all participants were tested anonymously makes it unlikely that they would be motivated to respond untruthfully to explicit ratings. Besides, our results seem to be consistent with a study that reports a decrease in implicit and an increase in explicit anti-fat/pro-thin attitudes over time in recent years (Tomiyama et al., 2015). If we assume that explicit attitudes are somehow reflective of the societal norm (Brewer and Brown, 1998), our results might be indicative that the current social view regarding anti-fat bias is more permissive (even that openly showing a dislike for fatness is socially acceptable) in the cultural context wherein this study was conducted. In any case, this reasoning is speculative and further research is needed.

As with the IRAP, the key between-group difference in explicit weight bias appears to be on the evaluation of fatness, with highly dissatisfied women showing significantly stronger explicit anti-fat attitudes. However, there was no significant implicit-explicit correlation in attitudes to fatness. Interestingly, both components were positively (and independently) correlated with self-report measures of BD, eating disorder symptoms, and body-image psychological inflexibility. Regression analyses showed that both types of attitudes were independent predictors of self-report body image measures, with contributions of similar magnitude to explained variance. Although the same target pictures and evaluative labels were used for the assessment of both types of attitudes, both procedures (IRAP and VAS) captured relevant differences between the immediate, automatic and the extended, elaborate verbal responses to fatness. This has potential relevant clinical implications. Most clinical studies typically use questionnaires and interviews that assess explicit beliefs about body image, rather than implicit beliefs. As suggested by our findings, the IRAP can provide added value in the prediction of clinically relevant body-image measures. In our opinion, this would justify its use in future studies with clinical population, or in prospective studies in order to isolate the relative contribution of implicit attitudes to predicting behavioral outcomes related to eating disorders and response to their treatment.

One potential limitation in this study is the attrition rate in the IRAP. 44% of participants who started the IRAP did not complete the task or produce valid data. It is worth noting that strict criteria were set not only for passing from the practice to the test phase, but also for consideration of test phase data as valid. Only the data from participants who maintained the criteria in all six test blocks were considered for analyses. Although it might have been possible to have a larger sample by setting less stringent latency and accuracy criteria as in other studies (e.g., Juarascio et al., 2011), or by removing only the pair of blocks where a participant failed to maintain the criteria instead of the whole dataset for that specific participant (e.g., Ritzert et al., 2016), we preferred to adhere to strict criteria in order to guarantee that the analyzed data were meaningful and reliable in terms of the briefness and immediacy of the captured responses (see Hughes et al., 2011). In any case, as mentioned in the Method section, the final sample in the study (*n* = 52) fulfills sample-size recommendations (Vahey et al., 2015). Another potential limitation concerns the set of photographic stimuli employed. Although every effort was made to insure that photos were discriminable only on the basis of body shape and not on other features (e.g., attractiveness), we did not conduct a validation procedure for the stimulus set.

Overall, the findings in this study point out the importance of taking into account variables like body dissatisfaction in research on implicit body-shape attitudes. Also, in terms of applied relevance, they suggest that prevention efforts for eating disorders should focus in promoting openness to and acceptance of a broader range of female bodies. It seems that having an implicit positive view of thinness is common across different degrees of BD and not really associated to body-image related distress or eating disorder symptoms. Also, a less positive implicit attitude toward fatness is associated to a higher level of BD and to clinically relevant measures of body image distress. Accordingly, it seems that the focus of prevention should not be about changing relations among thinness and desired outcomes (beauty, success, etc.), but about promoting the appreciation of larger body shapes through exposure to a diversity of women as instances of attractiveness, beauty and success.

## Data Availability

The datasets generated for this study are available on request to the corresponding author.

## Ethics Statement

All procedures in this study were in accordance with the institutional Ethics Board for Human Research, and with the Declaration of Helsinki 1964 and its later amendments or comparable ethical standards. Informed consent was obtained from all individual participants.

## Author Contributions

MH-L and MR-V conceived and designed the study, performed the statistical analysis, interpreted the results, and wrote the manuscript. AA-R recruited the participants, collected the data, and organized the database. All authors contributed to the manuscript revision, and read and approved the submitted version.

## Conflict of Interest Statement

The authors declare that the research was conducted in the absence of any commercial or financial relationships that could be construed as a potential conflict of interest.
